# Ethyl 3-carb­oxy-5-nitro­benzoate

**DOI:** 10.1107/S160053680901558X

**Published:** 2009-04-30

**Authors:** Ya-Ling Liu, Pei Zou, Min-Hao Xie, Shi-Neng Luo, Yong-Jun He

**Affiliations:** a Jiangsu Institute of Nuclear Medicine, Wuxi 214063, People’s Republic of China

## Abstract

In the title compound, C_10_H_9_NO_6_, the carb­oxy, ethoxy­carbonyl and nitro groups form dihedral angles of 3.8 (1), 4.5 (1) and 164.8 (1)°, respectively, with the mean plane of the benzene ring. In the crystal structure, mol­ecules lying about inversion centers are linked through O—H⋯O hydrogen bonds. C—H⋯O inter­actions are also present.

## Related literature

The title compound is an important inter­mediate for the preparation of iodinated X-ray contrast media, such as iotalamic acid, ioxitalamic acid, and ioxilan, which are used clinically all over the world (Morin *et al.*, 1987 ▶; Singh & Rathore, 1980 ▶; Stacul, 2001 ▶). For a related structure, see: Zou *et al.* (2009 ▶).
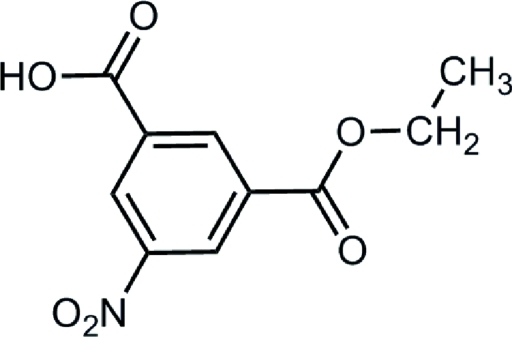


## Experimental

### 

#### Crystal data

C_10_H_9_NO_6_
*M* *_r_* = 239.18
Monoclinic, 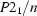

*a* = 14.249 (3) Å
*b* = 4.6450 (9) Å
*c* = 16.536 (4) Å
β = 108.401 (3)°
*V* = 1038.5 (4) Å^3^
*Z* = 4
Mo *K*α radiation
μ = 0.13 mm^−1^
*T* = 93 K
0.40 × 0.23 × 0.23 mm

#### Data collection

Rigaku SPIDER diffractometer
Absorption correction: none
6542 measured reflections
2355 independent reflections
1967 reflections with *I* > 2σ(*I*)
*R*_int_ = 0.024

#### Refinement

*R*[*F* ^2^ > 2σ(*F* ^2^)] = 0.043
*wR*(*F* ^2^) = 0.087
*S* = 0.99
2355 reflections
159 parameters
H atoms treated by a mixture of independent and constrained refinement
Δρ_max_ = 0.54 e Å^−3^
Δρ_min_ = −0.21 e Å^−3^

### 

Data collection: *RAPID-AUTO* (Rigaku, 2004 ▶); cell refinement: *RAPID-AUTO*; data reduction: *RAPID-AUTO*; program(s) used to solve structure: *SHELXS97* (Sheldrick, 2008 ▶); program(s) used to refine structure: *SHELXL97* (Sheldrick, 2008 ▶); molecular graphics: *SHELXTL* (Sheldrick, 2008 ▶); software used to prepare material for publication: *SHELXTL*.

## Supplementary Material

Crystal structure: contains datablocks I, global. DOI: 10.1107/S160053680901558X/pv2153sup1.cif

Structure factors: contains datablocks I. DOI: 10.1107/S160053680901558X/pv2153Isup2.hkl

Additional supplementary materials:  crystallographic information; 3D view; checkCIF report

## Figures and Tables

**Table 1:** Hydrogen-bond geometry (Å, °)

*D*—H⋯*A*	*D*—H	H⋯*A*	*D*⋯*A*	*D*—H⋯*A*
O5—H5*O*⋯O6^i^	0.92 (3)	1.71 (3)	2.630 (2)	176.7 (17)
C6—H6⋯O2^ii^	0.95	2.35	3.280 (2)	165
C9—H9*A*⋯O6^iii^	0.98	2.56	3.354 (3)	138

Symmetry codes: (i) 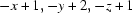; (ii) 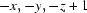; (iii) 
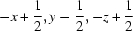
.

**Fig. 1.**
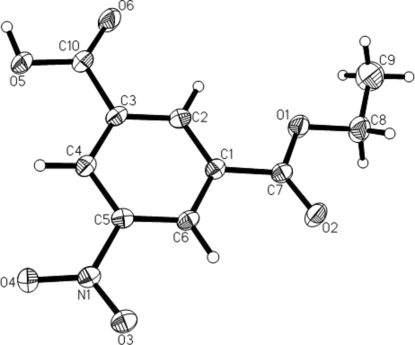
A view of the title compound with the atomic numbering scheme. Displacement ellipsoids were drawn at the 30% probability level.

## supplementary crystallographic information

### Comment

The title compound is an important intermediate for the preparation of iodinated X-ray contrast media, such as iotalamic acid, ioxitalamic acid, and ioxilan, which are used clinically all over the world (Morin *et al.*, 1987; Singh *et al.*, 1980; Stacul, 2001). We report here the crystal structure of the title compound.

The crystal data show that the bond lengths and angles in the title compound (Fig. 1) are within expected ranges and agree well with the corresponding molecular dimensions reported for a similar compound (Zou *et al.*, 2009). The carboxylic acid group (O5/C10/O6) attached at C3 and the ester group (O1/C7/O2) attached at C1 are nearly coplanar with the benzene ring (C1—C6) (dihedral angles of 3.8 (1) and 4.5 (1)°, respectively), while the nitro group (O3/N1/O4) attached at C5 forms a dihedral angle of 164.8 (1) ° with the benzene ring. In the cyrstal structure, the molecules lying about inversion centers are linked through O—H···O hydrogen bonds (Table 1).

### Experimental

5-Nitroisophthalic acid (2.1 g, 0.01 mol) was dissolved in 1.5 M ethanolic hydrochloric acid solution (7.5 ml) at 323 K. The mixture was stirred at 323 K for 6 hr. Then sodium chloride (1.8 g, 0.03 mol) in water (20 ml) was added. An oily liquid separated and crystallized on cooling. The precipitate was suction filtered and washed with water until neutral. The solid was suspended in sodium bicarbonate (1.0 g, 0.01 mol) in water (10 ml) and the undissolved diester was filtered off. The filtrate was acidified with 1 M hydrochloric acid to a pH of 4. The precipitate was filtered and washed with cold water. The crude product was purified by recrystallization from ethanol (yield: 41%). Single crystals were grown by slow evaporation of a ethanol/water(*v*/*v* 1:1) solution: colourless prismatic crystals were formed after several days.

### Refinement

All the H atoms could have been discerned in the difference electron density maps. With the exception of the hydrogen belonging to the hydroxyl group of the carboxyl the H atoms were situated into the idealized positions and refined in riding motion approximation. The hydroxyl hydrogen was refined freely. The used constraints: C_aryl_—H = 0.95 Å, C_methyl_—H = 0.98 Å and C_methylene_—H = 0.99 Å, *U*_iso_(H) = 1.2*U*_eq_(C).

### Crystal data

**Table:**

C_10_H_9_NO_6_	*F*(000) = 496
*M**_r_* = 239.18	*D*_x_ = 1.530 Mg m^−^^3^
Monoclinic, *P*2_1_/*n*	Mo *K*α radiation, λ = 0.71073 Å
Hall symbol: -P 2yn	Cell parameters from 3010 reflections
*a* = 14.249 (3) Å	θ = 3.0–27.5°
*b* = 4.6450 (9) Å	µ = 0.13 mm^−^^1^
*c* = 16.536 (4) Å	*T* = 93 K
β = 108.401 (3)°	Prism, colorless
*V* = 1038.5 (4) Å^3^	0.40 × 0.23 × 0.23 mm
*Z* = 4	

### Data collection

**Table:**

Rigaku SPIDER diffractometer	1967 reflections with *I* > 2σ(*I*)
Radiation source: Rotating Anode	*R*_int_ = 0.024
graphite	θ_max_ = 27.5°, θ_min_ = 3.0°
ω scans	*h* = −18→16
6542 measured reflections	*k* = −5→5
2355 independent reflections	*l* = −21→20

### Refinement

**Table:**

Refinement on *F*^2^	Primary atom site location: structure-invariant direct methods
Least-squares matrix: full	Secondary atom site location: difference Fourier map
*R*[*F*^2^ > 2σ(*F*^2^)] = 0.043	Hydrogen site location: inferred from neighbouring sites
*wR*(*F*^2^) = 0.087	H atoms treated by a mixture of independent and constrained refinement
*S* = 0.99	*w* = 1/[σ^2^(*F*_o_^2^) + (0.018*P*)^2^ + 0.9*P*] where *P* = (*F*_o_^2^ + 2*F*_c_^2^)/3
2355 reflections	(Δ/σ)_max_ < 0.001
159 parameters	Δρ_max_ = 0.54 e Å^−^^3^
0 restraints	Δρ_min_ = −0.21 e Å^−^^3^

### Special details

**Table:**

Geometry. All e.s.d.'s (except the e.s.d. in the dihedral angle between two l.s. planes)are estimated using the full covariance matrix. The cell e.s.d.'s are takeninto account individually in the estimation of e.s.d.'s in distances, anglesand torsion angles; correlations between e.s.d.'s in cell parameters are onlyused when they are defined by crystal symmetry. An approximate (isotropic)treatment of cell e.s.d.'s is used for estimating e.s.d.'s involving l.s. planes.
Refinement. Refinement of *F*^2^ against ALL reflections. The weighted *R*-factor *wR* andgoodness of fit *S* are based on *F*^2^, conventional *R*-factors *R* are basedon *F*, with *F* set to zero for negative *F*^2^. The threshold expression of*F*^2^ > σ(*F*^2^) is used only for calculating *R*-factors(gt) *etc*. and isnot relevant to the choice of reflections for refinement. *R*-factors basedon *F*^2^ are statistically about twice as large as those based on *F*, and *R*-factors based on ALL data will be even larger.

### Fractional atomic coordinates and isotropic or equivalent isotropic displacement parameters (Å^2^)

**Table:**

	*x*	*y*	*z*	*U*_iso_*/*U*_eq_	
O1	0.13780 (9)	0.1168 (3)	0.34968 (7)	0.0298 (3)	
O2	0.03728 (8)	0.0126 (3)	0.42538 (7)	0.0276 (3)	
O3	0.08919 (8)	0.5402 (3)	0.69486 (7)	0.0264 (3)	
O4	0.18028 (8)	0.9233 (3)	0.71824 (7)	0.0254 (3)	
O5	0.42632 (9)	1.0386 (3)	0.57257 (8)	0.0295 (3)	
O6	0.41511 (9)	0.7677 (3)	0.45857 (8)	0.0372 (3)	
N1	0.14799 (9)	0.7034 (3)	0.67837 (8)	0.0204 (3)	
C1	0.16237 (11)	0.3639 (3)	0.47870 (9)	0.0191 (3)	
C2	0.24472 (11)	0.5011 (4)	0.46934 (10)	0.0212 (3)	
H2	0.2662	0.4570	0.4219	0.025*	
C3	0.29609 (11)	0.7032 (4)	0.52916 (10)	0.0207 (3)	
C4	0.26459 (11)	0.7719 (3)	0.59811 (10)	0.0202 (3)	
H4	0.2985	0.9114	0.6389	0.024*	
C5	0.18231 (11)	0.6311 (3)	0.60566 (9)	0.0186 (3)	
C6	0.13113 (11)	0.4266 (3)	0.54859 (9)	0.0188 (3)	
H6	0.0759	0.3304	0.5566	0.023*	
C7	0.10519 (11)	0.1465 (4)	0.41636 (9)	0.0207 (3)	
C8	0.08258 (14)	−0.0899 (5)	0.28589 (11)	0.0362 (5)	
H8A	0.0140	−0.0217	0.2590	0.043*	
H8B	0.0802	−0.2788	0.3129	0.043*	
C9	0.13330 (17)	−0.1176 (6)	0.22150 (14)	0.0530 (6)	
H9A	0.1328	0.0686	0.1935	0.064*	
H9B	0.0991	−0.2611	0.1789	0.064*	
H9C	0.2018	−0.1787	0.2491	0.064*	
C10	0.38474 (12)	0.8391 (4)	0.51701 (10)	0.0242 (4)	
H5O	0.4809 (18)	1.103 (5)	0.5597 (14)	0.064 (8)*	

### Atomic displacement parameters (Å^2^)

**Table:**

	*U*^11^	*U*^22^	*U*^33^	*U*^12^	*U*^13^	*U*^23^
O1	0.0321 (6)	0.0351 (7)	0.0270 (6)	−0.0129 (6)	0.0161 (5)	−0.0098 (5)
O2	0.0262 (6)	0.0316 (7)	0.0261 (6)	−0.0117 (5)	0.0098 (5)	−0.0031 (5)
O3	0.0269 (6)	0.0273 (7)	0.0288 (6)	−0.0054 (5)	0.0143 (5)	0.0005 (5)
O4	0.0235 (6)	0.0245 (6)	0.0282 (6)	−0.0031 (5)	0.0080 (5)	−0.0077 (5)
O5	0.0227 (6)	0.0336 (7)	0.0357 (7)	−0.0122 (5)	0.0140 (5)	−0.0083 (6)
O6	0.0312 (7)	0.0485 (9)	0.0394 (7)	−0.0200 (6)	0.0218 (6)	−0.0161 (6)
N1	0.0171 (6)	0.0220 (7)	0.0220 (6)	0.0000 (5)	0.0057 (5)	0.0014 (6)
C1	0.0181 (7)	0.0184 (8)	0.0202 (7)	−0.0002 (6)	0.0052 (6)	0.0026 (6)
C2	0.0193 (7)	0.0234 (9)	0.0219 (7)	−0.0006 (6)	0.0079 (6)	0.0019 (7)
C3	0.0158 (7)	0.0212 (8)	0.0248 (7)	−0.0020 (6)	0.0060 (6)	0.0030 (7)
C4	0.0171 (7)	0.0189 (8)	0.0229 (7)	−0.0010 (6)	0.0040 (6)	0.0014 (6)
C5	0.0176 (7)	0.0194 (8)	0.0191 (7)	0.0018 (6)	0.0062 (6)	0.0032 (6)
C6	0.0155 (7)	0.0188 (8)	0.0219 (7)	−0.0003 (6)	0.0056 (6)	0.0051 (6)
C7	0.0200 (7)	0.0217 (9)	0.0209 (7)	−0.0010 (6)	0.0074 (6)	0.0024 (6)
C8	0.0397 (10)	0.0404 (12)	0.0311 (9)	−0.0145 (9)	0.0147 (8)	−0.0134 (8)
C9	0.0542 (14)	0.0675 (17)	0.0422 (12)	−0.0140 (12)	0.0224 (10)	−0.0208 (11)
C10	0.0197 (8)	0.0254 (9)	0.0272 (8)	−0.0044 (7)	0.0070 (6)	−0.0007 (7)

### Geometric parameters (Å, °)

**Table:**

O1—C7	1.3320 (19)	C2—H2	0.9500
O1—C8	1.460 (2)	C3—C4	1.388 (2)
O2—C7	1.1986 (19)	C3—C10	1.481 (2)
O3—N1	1.2230 (17)	C4—C5	1.382 (2)
O4—N1	1.2242 (17)	C4—H4	0.9500
O5—C10	1.308 (2)	C5—C6	1.375 (2)
O5—H5O	0.92 (3)	C6—H6	0.9500
O6—C10	1.223 (2)	C8—C9	1.469 (3)
N1—C5	1.4726 (19)	C8—H8A	0.9900
C1—C2	1.386 (2)	C8—H8B	0.9900
C1—C6	1.394 (2)	C9—H9A	0.9800
C1—C7	1.488 (2)	C9—H9B	0.9800
C2—C3	1.392 (2)	C9—H9C	0.9800
			
C7—O1—C8	114.65 (13)	C5—C6—H6	120.8
C10—O5—H5O	107.3 (15)	C1—C6—H6	120.8
O3—N1—O4	124.39 (13)	O2—C7—O1	123.74 (15)
O3—N1—C5	117.92 (13)	O2—C7—C1	123.56 (14)
O4—N1—C5	117.69 (13)	O1—C7—C1	112.70 (13)
C2—C1—C6	119.97 (14)	O1—C8—C9	107.75 (16)
C2—C1—C7	122.15 (14)	O1—C8—H8A	110.2
C6—C1—C7	117.88 (14)	C9—C8—H8A	110.2
C1—C2—C3	120.29 (14)	O1—C8—H8B	110.2
C1—C2—H2	119.9	C9—C8—H8B	110.2
C3—C2—H2	119.9	H8A—C8—H8B	108.5
C4—C3—C2	120.32 (14)	C8—C9—H9A	109.5
C4—C3—C10	121.61 (14)	C8—C9—H9B	109.5
C2—C3—C10	118.07 (14)	H9A—C9—H9B	109.5
C5—C4—C3	117.97 (14)	C8—C9—H9C	109.5
C5—C4—H4	121.0	H9A—C9—H9C	109.5
C3—C4—H4	121.0	H9B—C9—H9C	109.5
C6—C5—C4	123.11 (14)	O6—C10—O5	123.54 (15)
C6—C5—N1	118.49 (13)	O6—C10—C3	121.42 (15)
C4—C5—N1	118.40 (14)	O5—C10—C3	115.04 (14)
C5—C6—C1	118.31 (14)		
			
C6—C1—C2—C3	−0.5 (2)	C2—C1—C6—C5	1.7 (2)
C7—C1—C2—C3	179.98 (14)	C7—C1—C6—C5	−178.76 (13)
C1—C2—C3—C4	−0.8 (2)	C8—O1—C7—O2	1.3 (2)
C1—C2—C3—C10	178.79 (14)	C8—O1—C7—C1	−178.77 (14)
C2—C3—C4—C5	0.9 (2)	C2—C1—C7—O2	175.54 (15)
C10—C3—C4—C5	−178.73 (14)	C6—C1—C7—O2	−4.0 (2)
C3—C4—C5—C6	0.4 (2)	C2—C1—C7—O1	−4.3 (2)
C3—C4—C5—N1	−179.71 (13)	C6—C1—C7—O1	176.11 (14)
O3—N1—C5—C6	14.8 (2)	C7—O1—C8—C9	−174.64 (17)
O4—N1—C5—C6	−165.20 (13)	C4—C3—C10—O6	176.32 (16)
O3—N1—C5—C4	−165.12 (14)	C2—C3—C10—O6	−3.3 (2)
O4—N1—C5—C4	14.9 (2)	C4—C3—C10—O5	−4.1 (2)
C4—C5—C6—C1	−1.7 (2)	C2—C3—C10—O5	176.29 (14)
N1—C5—C6—C1	178.44 (13)		

### Hydrogen-bond geometry (Å, °)

**Table:**

*D*—H···*A*	*D*—H	H···*A*	*D*···*A*	*D*—H···*A*
O5—H5O···O6^i^	0.92 (3)	1.71 (3)	2.630 (2)	176.7 (17)
C6—H6···O2^ii^	0.95	2.35	3.280 (2)	165
C9—H9A···O6^iii^	0.98	2.56	3.354 (3)	138

Symmetry codes: (i) −*x*+1, −*y*+2, −*z*+1; (ii) −*x*, −*y*, −*z*+1; (iii) −*x*+1/2, *y*−1/2, −*z*+1/2.
